# Genome-wide exploration of the GDSL-type esterase/lipase gene family in rapeseed reveals several *BnGELP* proteins active during early seedling development

**DOI:** 10.3389/fpls.2023.1139972

**Published:** 2023-03-15

**Authors:** Yahui Ding, Liwen Xing, Jiamin Xu, Teng Jiang, Xiuhua Tang, Yaxuan Wang, Shuhua Huang, Wenfang Hao, Xiaona Zhou, Yanfeng Zhang, Chang Gen Xie

**Affiliations:** ^1^ State Key Laboratory of Crop Stress Biology for Arid Areas, College of Life Sciences, Northwest A&F University, Yangling, China; ^2^ Hybrid Rapeseed Research Centre of Shaanxi Province, Yangling, China

**Keywords:** Gly-Asp-Ser-Leu (GDSL)-type esterases/lipases, GELP, lipid mobilization, *Brassica napus*, seed germination

## Abstract

The Gly-Asp-Ser-Leu (GDSL)-type esterase/lipase proteins (GELP) are one of the most important families of lipolytic enzymes and play prominent roles in seed germination and early seedling establishment through mobilizing the lipids stored in seeds. However, there are no comprehensive studies systematically investigating the *GELP* gene family in *Brassica napus* (*BnGELP*), and their biological significance to these physiological processes are far from understood. In the present study, a total of 240 *BnGELP* genes were identified in *B. napus* cultivar “Zhongshuang 11” (ZS11), which is nearly 2.3-fold more *GELP* genes than in *Arabidopsis thaliana*. The *BnGELP* genes clustered into 5 clades based on phylogenetic analysis. Ten BnGELPs were identified through zymogram analysis of esterase activity followed by mass spectrometry, among which five clustered into the clade 5. Gene and protein architecture, gene expression, and *cis*-element analyses of *BnGELP* genes in clade 5 suggested that they may play different roles in different tissues and in response to different abiotic stresses. *BnGELP99* and *BnGELP159* were slightly induced by cold, which may be attributed to two low-temperature responsive *cis*-acting regulatory elements present in their promoters. An increased activity of esterase isozymes by cold was also observed, which may reflect other cold inducible esterases/lipases in addition to the ten identified BnGELPs. This study provides a systemic view of the *BnGELP* gene family and offers a strategy for researchers to identify candidate esterase/lipase genes responsible for lipid mobilization during seed germination and early seedling establishment.

## Introduction

Rapeseed (*Brassica napus* L., genome AACC, 2n = 4x = 38) is the third most important oil crop across the world (Ding et al., 2019a), and is an allotetraploid derived from an interspecific hybridization between *Brassica rapa* (genome AA, 2n = 20) and *Brassica oleracea* (genome CC, 2n = 18), with subsequent chromosomal doubling (Sun et al., 2017; Chen et al., 2021). Seed oil content (OC), one of the most important economic and nutritional traits for rapeseed, is mainly determined by a dynamic balance between lipid synthesis and breakdown during seed maturation (Ding et al., 2019a; Wang et al., 2021). In rapeseed, lipid biosynthesis is initiated just after embryo formation, and its reduction and decomposition occurs during the late maturation stage (Wang et al., 2021). Oil bodies, or lipid droplets, are the main reservoir of lipids in seeds of *B. napus*.

While breeding for cultivars with high OC, seed germination and seedling development are also targeted traits due to their impact on successful propagation (Wang et al., 2021). In *B. napus*, seed germination and early development of the seedling are mainly dependent on the catabolism of stored lipids, which is initiated by the action of esterases/lipases (Clauss et al., 2008; Zhu et al., 2017; Ding et al., 2019a; Wang et al., 2021). GDSL-type esterases/lipases (GELPs) are one type of lipolytic enzyme that mobilizes the stored lipid in oil bodies during germination and early seedling establishment (Akoh et al., 2004; Clauss et al., 2008; Ding et al., 2019a; Ding et al., 2019b; Wang et al., 2021; Shen et al., 2022). GELPs are named after the highly conservative GDSL motif, GDSxxDxG, mainly present close to the N-terminus of the protein (Akoh et al., 2004; Ding et al., 2019b; Shen et al., 2022). GELPs usually harbor five conserved sequence blocks (I-V), which are also important for their classification (Akoh et al., 2004; Ding et al., 2019b; Shen et al., 2022). There are four strictly invariant residues present in each of the four of the conserved blocks, serine (S) in block I, glycine (G) in block II, asparagine (N) in block III and histidine (H) in block V (Akoh et al., 2004; Ding et al., 2019b; Shen et al., 2022). For this reason, GELPs are also called SGNH hydrolases (Akoh et al., 2004; Ding et al., 2019a; Ding et al., 2019b; Shen et al., 2022). GELPs are hydrolytic enzymes that display broad substrate specificity and regio-specificity. Therefore, it is easy to detect the biochemical activity of different GELPs present in a total protein extract from seedlings in a polyacrylamide gel, such as through determining the zymogram of esterase/lipase isozymes (Akoh et al., 2004; Zhu et al., 2017; Ding et al., 2019b; Shen et al., 2022).

GELPs are widely distributed in microbes, animals and plants (Akoh et al., 2004). GELPs have been suggested to play significant roles in controlling nearly all aspects of plant growth and development as well as response to abiotic and biotic stresses (Shen et al., 2022). A crucial first step in transferring the knowledge acquired for model species to non-model species is homology identification. To date, the *GELP* family has been identified in diverse plant species including many with fully sequenced genomes, such as *Arabidopsis thaliana* (105 members)*, Dasypyrum villosum* (193)*, Sedum alfredii* (80), *Nicotiana tabacum* (159), *Glycine max* (194)*, Oryza sativa* (114)*, Dendrobium catenatum* (52), *Carya illinoensis* (87)*, Zea mays* (103)*, Vitis vinifera* (83), *Brassica rapa* (121), and six species in the Rosaceae family (*Pyrus bretschneideri*, *Malus domestica*, *Prunus mume*, *Prunus persica*, *Fragaria vesca* and *Prunus avium*; for a total of 597 *GELP*s) (Chepyshko et al., 2012; Dong et al., 2016; Lai et al., 2017; Ding et al., 2019b; Su et al., 2020; Lv et al., 2021; Wang et al., 2021; Zhang et al., 2021; Cenci et al., 2022; Jiao et al., 2022; Shen et al., 2022). Some *GELP* genes have been cloned and functionally characterized in *B. napus*, such as *BnLIP2* (Ling et al., 2006), *Bn*SCE3 (Clauss et al., 2008), *BnGDSL1* (Ding et al., 2019a), and *BnSFAR1*-*5* (Karunarathna et al., 2020). However, no comprehensive study aiming to systematically investigate the *GDSL* gene family has been conducted in *B. napus*, particularly in cultivar ZS11, for which a high-quality genome assembly has been recently released (Chen et al., 2021). ZS11 is a cultivar with stable yield and its recently published genome assembly is more accurate and complete than the previous genomes (Chalhoub et al., 2014; Bayer et al., 2017; Sun et al., 2017), providing an excellent opportunity for reliable, systematic exploration of all the genes belonging to a specified family.

In the current study, a total of 240 *GDSL-*like genes were identified from the genome of *B. napus* cultivar ZS11. A systematic analysis of the *BnGDSL *gene family, including phylogenetic relationships, chromosomal location, gene synteny, gene and protein structure, including the *cis*-elements present in the promoters, and expression profiles in different tissues and in response to different abiotic stresses was conducted to gain insights into the evolutionary and functional characteristics of the *BnGELP* gene family.

## Materials and methods

### Identification of the *GELP* gene family in *Brassica napus*

Protein sequences of the *B. napus* cultivar (ZS11) were download from the National Genomics Data Center (NGDC) (accession number: PRJCA002883) (Chen et al., 2021), and a local protein database was established using Hidden Markov model (HMM) profiles as seeds. The putative GELP protein family in *B. napus* (BnGELP) was predicted from ZS11 with the HMM profile corresponding to the Pfam GDSL domain (PF00657) by running the HMMER 3.0 software (with E-value < e^–10^). The preliminarily searched candidate protein sequences were then submitted to the Pfam website, the NCBI-CDD server (http://www.ncbi.nlm.nih.gov/Structure/cdd/wrpsb.cgi) and the SMART (Simple Modular Architecture Research Tool, http://smart.embl-heidelberg.de/) database for GDSL domain verification as previously described (Song et al., 2022).

### Analysis of predicted *GELP* proteins

The full-length protein sequences of the GELPs from *B. napus* cultivar ZS11 and *A. thaliana* were aligned by the MUSCLE tool, and a phylogenetic tree was generated using MEGA 11 with the neighbor-joining method (bootstrap replications, n=1000). Visualization of the phylogenetic tree was performed with FigTree v1.4.4 software. The online program EXPASY (https://web.expasy.org/protparam/) was used to calculate the molecular weight, theoretical isoelectric point (pI) and instability index of the BnGELP protein family members. The online software Wolfpsort (https://wolfpsort.hgc.jp/) was used for signal peptide and subcellular localization prediction of the BnGELP proteins. Conserved motifs and protein architecture were predicted by the on-line tool MEME (Multiple Em for Motif Elicitation, https://meme-suite.org/) and visualized by TBtools software (Bai et al., 2016; Chen et al., 2020).

### Genome-scale synteny analysis of *GELP* genes

All 240 BnGLEP and 105 AtGELP protein sequences were aligned using BLASTP with a threshold value of e^–10^. Duplications of the *BnGELP* genes were determined using BLAST and MCScanX with default parameters, and divided into tandem and segmental duplications as previously reported (Chen et al., 2020; Song et al., 2022). Similarly, the synteny between the *AtGELP* and *BnGELP* genes were also examined using TBtools. Collinearity diagrams were drawn as previously reported (Chen et al., 2020; Song et al., 2022).

### Chromosome localization and gene structure analyses

The physical positional information of each gene (GFF file and genome file) in *B. napus* cultivar ZS11 was downloaded from NGDC (accession number: PRJCA002883). Distribution of the *BnGELP* genes on the 19 chromosomes of *B. napus* were then deciphered with Mapchart, as previously described (Chen et al., 2020). The predicted introns and exons of the clade 5 *BnGELP* genes were extracted from the GFF file, and their intron/exon structures were drawn using Gene Structure View (Advanced) from TB tools as previously described (Song et al., 2022).

### Plant growth, cold treatment and seed oil content measurement

Seeds of *B. napus* cultivar ZS11 and inbred lines C69, A260, Y196, C145, GH124 and Y172 were placed on petri dishes covered with two layers of filter paper, and grown at 24°C. The whole seedlings were harvested when they were around 5 cm height and stored at -80°C. A set of seedlings of ZS11 were cold treated at 4°C and harvested after 0, 12, 24, 36, 48, 60 and 72 hours.

Mature seeds of *B. napus* cultivar ZS11 and inbred lines C69, A260, Y196, C145, GH124 and Y172 were harvested. Seed oil content (OC) was determined with near-infrared spectroscopy (ANTARIS II, Thermo Scientific™, USA) as previously described (Wang et al., 2021).

### Esterase isozyme electrophoresis and mass spectrometry analysis

Seedlings from ZS11 and the inbred lines were ground into homogenate in an ice-water bath with extraction buffer (100 mM Tris-HCl pH 8.0). The homogenate was then centrifuged at 8000 r/min for 10min. The supernatant was transferred into new tubes. Around 20 μg extracted protein was separated by native polyacrylamide gel electrophoresis (resolving gel 8%, stacking gel 3.6%). After electrophoresis, the gel was first stained with an esterase developing solution (an ethanol solution containing 1% α- naphthyl acetate and 2% β- naphthyl mixed with 2% naphtanil diazo blue B dissolved in 50 mM phosphate buffer at pH 6.4). The gel was destained, fixed with 2% acetic acid after each esterase isozyme band of the zymogram was observed and photographed as previously described (Van Xuan and Jin, 2011; Zhu et al., 2017). Each esterase isozyme band in region I of the zymogram was cut and destained. The protein bands were treated with 100 mM DTT and 60 mM iodoacetamide (IAM) and then digested with trypsin overnight at 37°C. The peptides were collected and desalted using C18 tips. The resulting peptides were analyzed by high-sensitivity LC-MS/MS (QExactive HF-X, ThermoFisher, Waltham, USA) for protein identification using Thermo Scientific Proteome Discoverer 2.4 software as previously described (Wang et al., 2022).

### Expression profiles of *BnGELP* genes determined by transcriptome data

RNA-seq data from 12 tissues (root, stem, leaf, flower, silique, sepal, pistil, stamen, ovule, pericarp, wilting pistil, and blossomy pistil) and under abiotic stresses (dehydration, NaCl, ABA and cold conditions) of ZS11 were downloaded from NGDC (accession numbers PRJNA394926 and CA001775) (Sun et al., 2017; Chen et al., 2021). The FastQC tool was used to check the data quality. Trimmomatic (-0.39) was used to filter the data. All of these RNA-seq data were mapped to the reference genome of ZS11 with HISAT2 2.04 software as previously described (Song et al., 2022). Transcript levels of the *BnGELP* genes were calculated using transcripts per kilobase of exon model per million mapped reads (TPM) values with the StringTie plug-in in TBtools. A histogram was generated *via* TBtools software as previously described (Song et al., 2022).

### Identification of *cis*-elements in the promoters of the clade 5 *BnGELP* genes

To *cis*-regulatory elements prediction, 2-kb sequences upstream from the start codon of each clade 5 *BnGELP* gene were extracted from the *B. napus* cultivar ZS11 genome database. The *cis*-regulatory elements in the promoters were predicted with the online website PlantCARE (http://bioinformatics.psb.ugent.be/webtools/plantcare/html/). The obtained results were filtered, categorized, and then visualized using TBtools as previously described (Li et al., 2019; Su et al., 2020).

### Cold-reponsive expression of *BnGELP* genes encoding the proteins identified by mass spectrometry

The expression of the *BnGELP* genes encoding the proteins from the zymograms and identified by mass spectrometry were determined in response to cold. One-week-old seedlings were moved to a chamber held 4 °C and sampled at 0, 12, 24, 36, 48, 60 and 72 hours. The total RNA was extracted with TRIzol reagent (TaKaRa). The complementary DNA (cDNA) was obtained after reverse transcription with PrimeScript™ RT Master Mix (Perfect Real Time, TaKaRa). Semi-quantitative RT-PCR was performed and normalized to ZS11C02G003910, which is an *actin*-like gene in *B. napus*, as previously described (Bai et al., 2016; Li et al., 2018).

## Results

### Genome-wide identification of *BnGELP* genes

The HMMER search found 240 putative BnGELP proteins with conserved GDSL-like lipase domains from the genome of *B. napus* cultivar ZS11, with 119 *BnGELP* genes encoded in the A subgenome and 121 genes in the C subgenome (**Supplementary Table 1**). This number of BnGELP proteins is around 2.3-fold more than encoded in *A. thaliana*. The *BnGELPs* were named in terms of their location on the chromosomes (**Supplementary Table 1**). The protein length of the predicted BnGELPs ranged from 158 to 1400 amino acids (aa), with predicted molecular weights (Mw) from 17.16 to 154.33 kDa. The theoretical isoelectric points (pI) ranged from 4.67 to 10.11 (**Supplementary Table 1**). The isoelectric points of 140 of the BnGELPs (58.33%) were greater than 7. Other characteristics of the BnGELPs, such as gene position, predicted subcellular localization, instability index and accession number, are presented in **Supplementary Table 1**.

### Classification and phylogenetic analyses of *BnGELP* proteins

**Figure 1:**
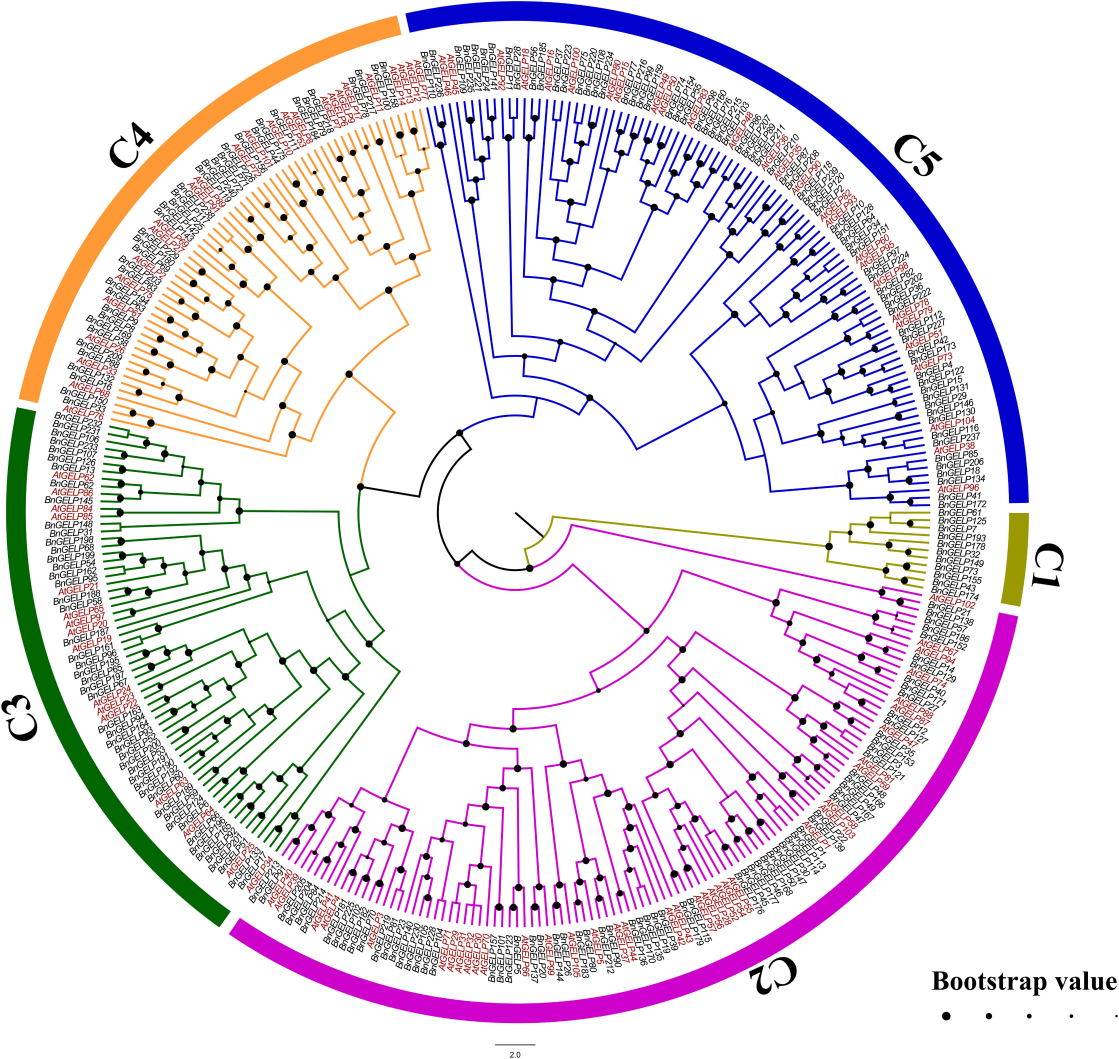
Phylogenetic analysis of GSDL-type Esterases/Lipases from *B*. *napus* and *A*. *thaliana.* The sequences of 240 BnGELP and 105 AtGELP proteins were aligned by the MUSCLE tool. A phylogenetic tree was generated using MEGA 11 with the neighbor-joining (NJ) method (bootstrap replications, n=1000). The phylogenetic tree was highlighted with FigTree (version 1.4.4). The proteins are clustered into 5 distinct clades designated with a clade number (C1~C5) and labelled with different colors.

**Figure 2:**
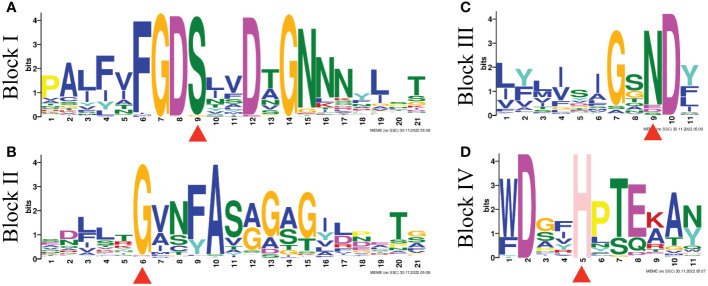
GDSL motif logos of four conserved blocks found in BnGELP proteins. The motif logogram was determined using MEME. The conserved residues Ser, Gly, Asn, and His are in blocks I **(A)**, II **(B)**, III **(C)** and V **(D)** all BnGELP proteins and are indicated with red triangles.

To explore the phylogenetic relationships of the GELPs in *A. thaliana* and *B. napus*, the protein sequences of 240 BnGELPs and 105 AtGELPs were aligned by the MUSCLE tool, and a phylogenetic tree was generated using MEGA 11 with the neighbor-joining method. As shown in **Figure 1**, the BnGELP proteins were classified into 5 clades, including 11 in clade 1, 72 in clade 2, 49 in clade 3, 38 in clade 4 and 70 in clade 5 (**Supplementary Table 2**). The four strictly invariant residues in the blocks of the GDSL motif were present in almost all 240 BnGELP proteins (**Figure 2**). Clades 2 (72) and 5 (70) were the two largest, and together represented around 60% of all BnGELP family members. In contrast, clade 1 had the fewest members (**Supplementary Table 2**).

### Chromosomal distribution of *BnGELP* genes

The 240 *BnGELP* genes were mapped onto the 19 chromosomes of *B. napus* (**Supplementary Figure 1**). There were 119 *BnGELP* genes anchored on chromosomes A01 to A10 and 121 *BnGELP* genes on chromosomes C01 to C09. The *BnGELP* genes were unevenly scattered on each chromosome at different distribution densities. The chromosomes C03 (21 members), C06 (20 members) and C02 (19 members) had a relatively higher density of *BnGELP* genes, while chromosomes C08 (4 members), A10 (5 members) and C01 (6 members) had fewer *BnGELP* genes (**Supplementary Figure 1** and **Supplementary Table 1**).

### Gene duplication and collinearity survey of the *BnGELP* genes

**Figure 3:**
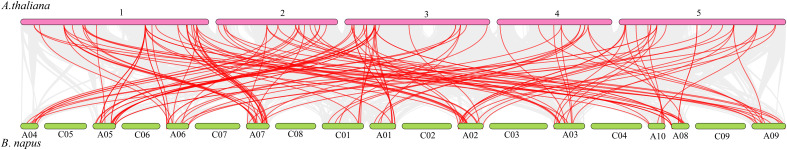
Synteny evaluation of *AtGELP* and *BnGELP* genes. The gray lines indicate all collinear blocks, and the red lines highlight the orthologous relationships between *AtGELPs* and *BnGELPs*.

To ascertain the evolution of the *BnGELP* genes, we first determined the synteny between *B. napus* and *A. thaliana* at the whole genome level. A total of 138 collinear gene pairs were observed between the two genomes (**Figure 3** and **Supplementary Table 3**). Most (36 out of 73) *AtGELP* genes had multiple syntenic genes in *B. napus*. For instance, both *AtGELP22* (At1g54000) and *AtGELP23* (At3G14210) had 6 collinear *BnGELP* genes. However, there were 37 *AtGELP* genes that had only one syntenic *BnGELP* gene (**Supplementary Table 4**).

To gain an insight into the synteny, we determined the putative tandem and segmental duplication events of the *BnGELP* family members using BLAST and MCScanX. A total of 2 tandem duplication events and 280 segmental duplications were observed in the *B. napus* genome (**Supplementary Figure 2** and **Supplementary Table 3**). Only 9 segmental duplication events were observed within the same chromosome, and three of them were observed on chromosome C06. In total, 271 segmental duplications were detected across the chromosomes (**Supplementary Table 3**). Further analysis revealed that there were 47 duplication events within the A subgenome, 48 events within the C subgenome, and 187 events between the A and C subgenomes (**Supplementary Table 3**). In terms of the U triangle, allopolyploidization may have played a role in the expansion of the *BnGELPs* gene family in *B. napus*.

### Several *BnGELP* proteins are related to esterase isozyme activity during early seedling development

**Figure 4:**
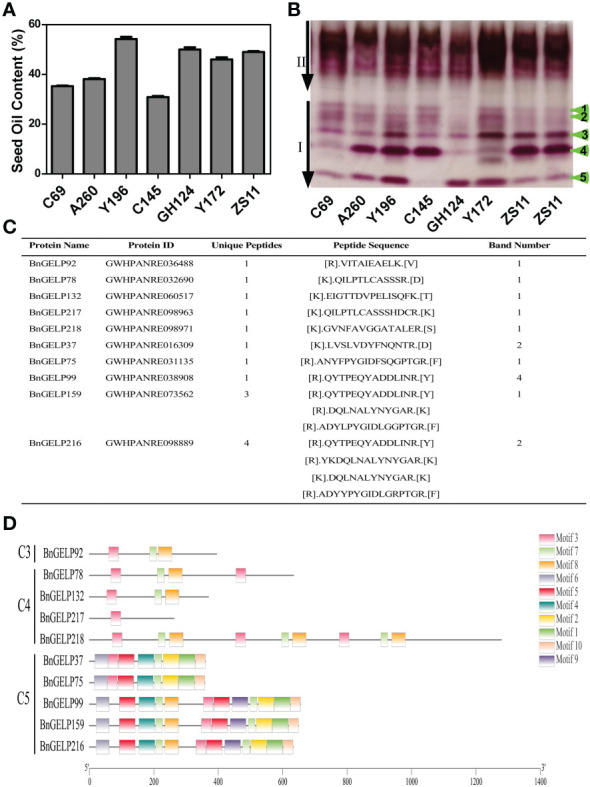
A zymogram of esterase isozymes in different *B*. *napus* materials. The seeds were from cultivar ZS11and inbred lines C69, A260, Y196, C145, GH124 and Y172. **(A)** Seed oil content of the *B*. *napus* materials. **(B)** The zymogram of esterase isozymes in different *B*. *napus* materials. **(C)** Mass spectrometric identification of different bands in region I of the zymogram in **(B)**. Selected unique proteins and the corresponding sequences are indicated. **(D)** Conserved motifs of the identified BnGELP proteins from region I of the zymogram. Boxes of different colors represent different motifs.

The level of oil content in *B. napus* is generally correlated with the dynamic balance between lipid biosynthesis and metabolism (Wang et al., 2021). Esterases or lipases are involved in hydrolyzing the stored triacylglycerols and converting them into hexoses and sucrose for the establishment of seedlings (Ding et al., 2019a; Wang et al., 2021). To explore the putative interrelationships between oil content and esterase isozyme activity, we electrophoresed the enzyme extracts from the seeds of different *B. napus* cultivars and inbred lines with distinct oil content profiles (**Figure 4**). There were several differences in region I of the zymogram among the different lines. The banding patterns of two high oil content (OC) inbred lines, Y172 (approximately 47% OC) and GH124 (approximately 51% OC), were different than the zymogram of cultivar ZS11 (approximately 49% OC). In contrast to ZS11, GH124 had weak third and fourth bands in region I. For Y172, the fourth band in region I was weak, while the first and second bands were stronger than in ZS11 and GH124 (**Figure 4B**). Moreover, three inbred lines with low oil content, C69 (approximately 35% OC), A260 (approximately 38% OC) and C145 (approximately 30% OC), also had different banding patterns in the zymogram compared to cultivar ZS11. Compared with ZS11, the third and fourth bands in region I were weak in C69, A260 and C145. However, the first and second bands in C69, A260 and C145 were stronger than in ZS11. Surprisingly, the fifth band in region I was not observed in C145 (**Figure 4B**).

To examine the possible esterases or lipases related to the observed differences in the zymogram, the five indicated bands were cut from the polyacrylamide gel for identification of the proteins *via* mass spectrometry. Since there are many esterases or lipases associated with lipid metabolism during germination, we performed Gene Ontology (GO) enrichment analysis of the complete portfolio of the identified proteins with respect to esterases or lipases (**Supplementary Table 5**). One clade 3 BnGELP protein (BnGELP92), four clade 4 BnGELP proteins (BnGELP78, 132, 217 and 218), and five clade 5 BnGELP proteins (BnGELP37, 75, 99, 159 and 216) were identified by at least one unique peptide (**Figures 4C, D** and **Supplementary Tables 5**, **6**). The identified BnGELP proteins falls into three major bands in region I of the zymogram: six were identified from the first band (BnGELP37, 75, 78, 159, 217 and 218), two from the second band (BnGELP37 and 216), and one from the fourth band (BnGELP99, **Figure 4C** and **Supplementary Table 5**).

### Gene and protein structure analyses of *BnGELP*s in the clade 5

**Figure 5:**
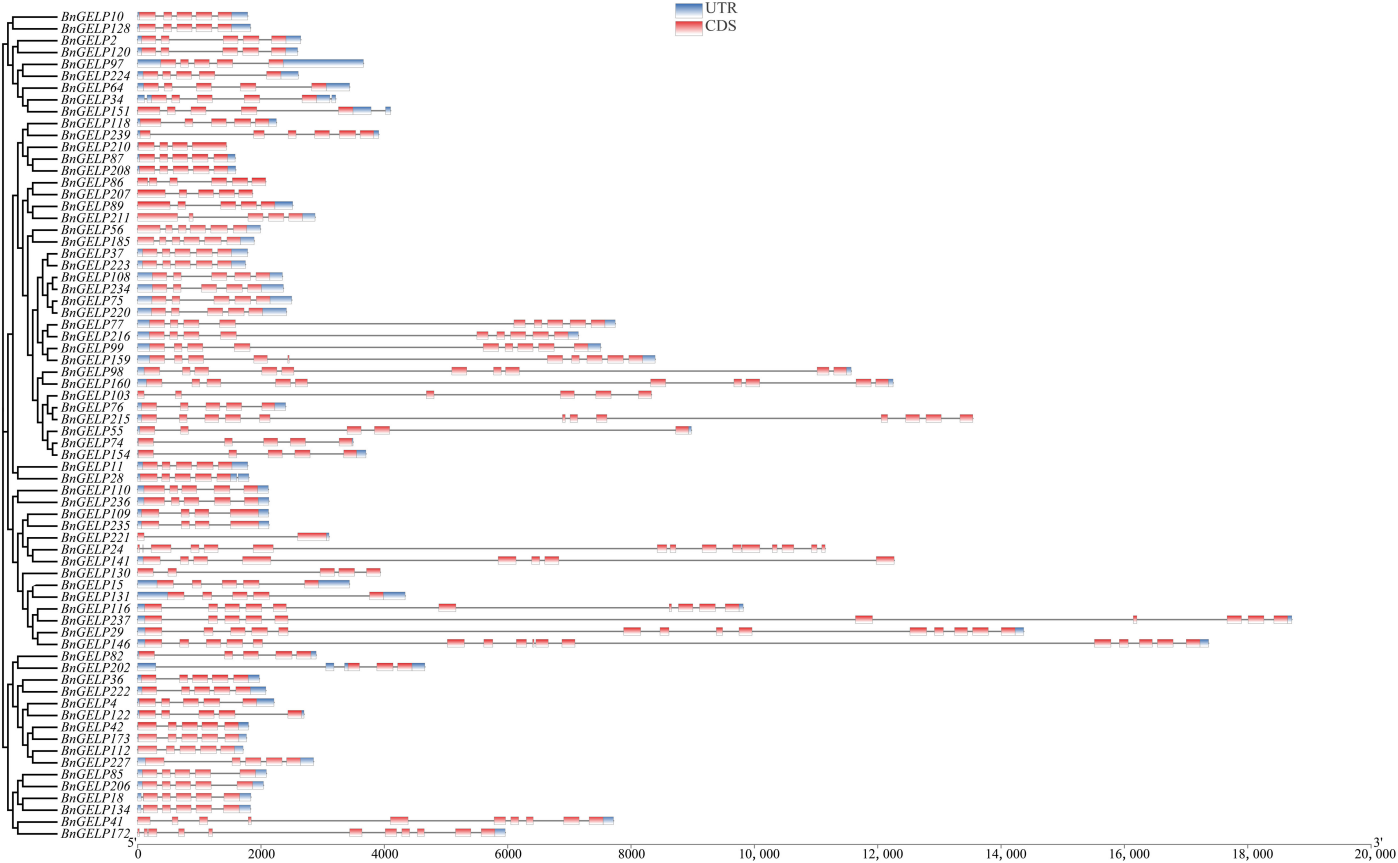
Schematic exon/intron structures of the clade 5 *BnGELP* genes. The red boxes represent exons and black lines represent introns. The UTR regions are indicated in blue boxes. The length of each CDS can be estimated by the scale at the bottom.

Since five of the ten identified BnGELP proteins in region I of the zymogram were from clade 5 (**Figure 4** and **Supplementary Figure 3**), we chose to systemically analyze the 70 proteins in this clade. We first analyzed the chromosomal localization of the clade 5 *BnGELP* genes. Among the 70- *BnGELP* genes of clade 5, 36 were encoded on the A subgenome chromosomes and 34 on the C subgenome chromosomes (**Supplementary Figure 1**). We also determined the overall exon/intron profile of the *BnGELP* genes within clade 5 (**Figure 5**). All *BnGELP* genes were interrupted by at least one intron. *BnGELP146* contained the most introns (15 introns), while *BnGELP221* had the fewest (one intron). More than 58% of the *BnGELP* genes (41 genes) in this clade were interrupted by four introns and composed of five exons in their coding regions. Paralogous genes in the phylogenetic tree often had similar gene structureS (**Figure 5**). All four members of the *BnGELP159* subclade (*BnGELP77, 216, 99* and *159*), two members of the *BnGELP37* subclade (*BnGELP37* and *223*) and four members of the *BnGELP75* subclade (*BnGELP75, 108, 220* and *234*) showed highly similar exon/intron arrangements (**Figure 5**). We also examined the protein architecture of the five identified BnGELP proteins in clade 5 (BnGELP37, 75, 99, 159 and 216). Consistent with the gene structure, almost all of the homologous proteins in the same subclade displayed similar arrangement of protein motifs (**Supplementary Figures 4**, **5** and **Supplementary Table 7**).

### Expression patterns of the clade 5 *BnGELP* genes across different tissues

**Figure 6:**
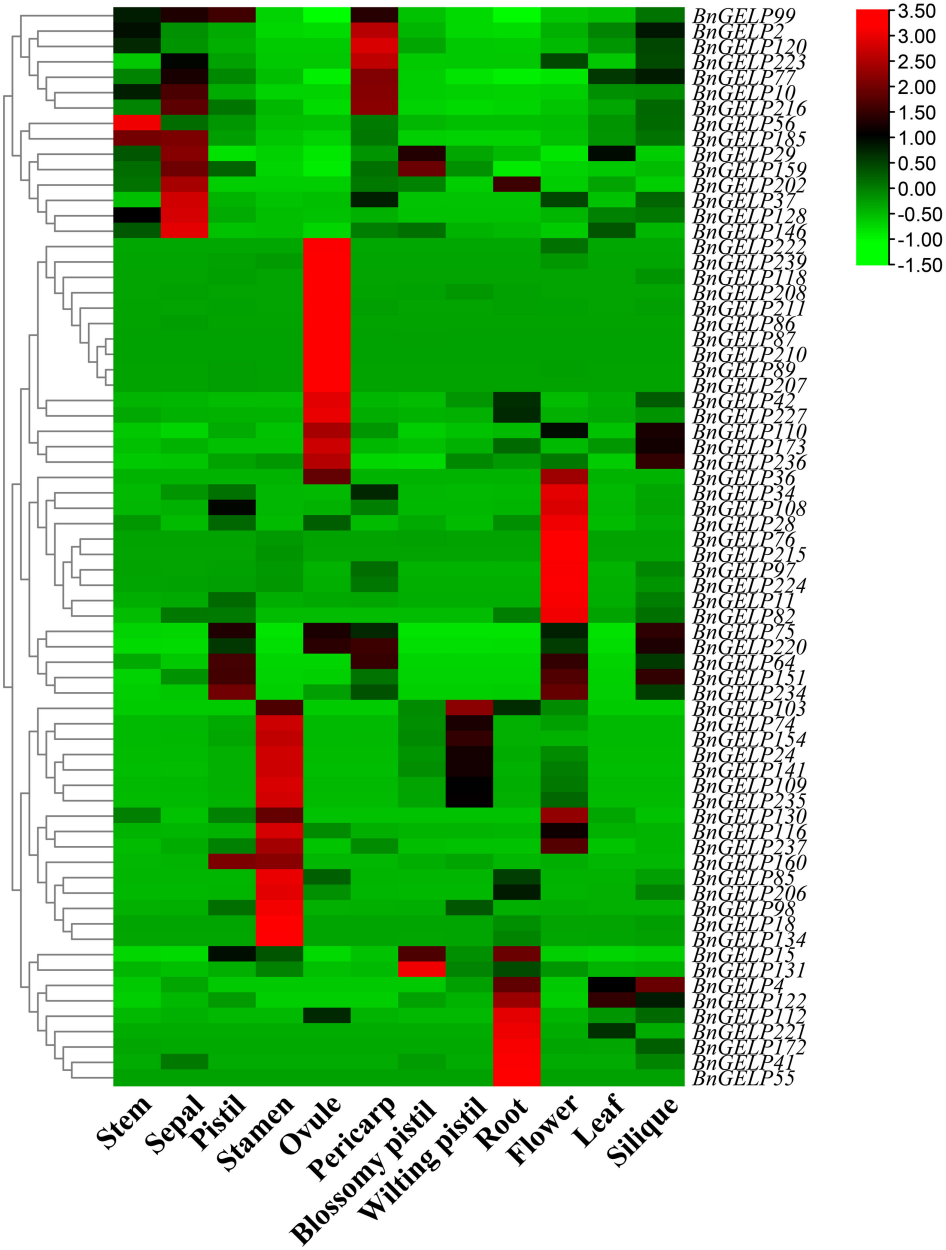
Expression profiles of the clade 5 *BnGELP* genes in different tissues. Transcript levels for the 70 *BnGELP* genes were obtained from public transcriptome data obtained from different tissues of *B*. *napus* cultivar ZS11. Each relative expression level is equal to the mean values and transformed log_2_ values for normalization. The color scale represents relative expression levels from low (green colored) to high (red colored).

The expression profiles of all 70 *BnGELP* genes in clade 5 were determined in 12 different tissues (stem, sepal, pistil, stamen, ovule, pericarp, blossomy pistil, wilting pistil, root, flower, leaf and silique; **Supplementary Figure 6**), using publicly available RNA-seq data from ZS11 plants (Song et al., 2022). All genes were detected in at least one tissue (**Figure 6** and **Supplementary Table 8**). The gene *BnGELP55* was specifically expressed in root, whereas 15 genes were detected in all of the analyzed tissues (**Figure 6** and **Supplementary Table 8**). Forty-nine genes were detected in leaf. The expression of the five identified *BnGELP* genes in clade 5 (*BnGELP37, 75, 99, 159* and *216*) was diverse. *BnGELP159* displayed higher expression levels in sepal and blossomy pistil, while its close paralogs, *BnGELP99* and *216*, showed higher levels in pistil and pericarp, respectively (**Figure 6** and **Supplementary Table 8**). *BnGELP37* displayed higher expression levels in sepal, while *BnGELP75* showed higher levels in pistil and silique (**Figure 6** and **Supplementary Table 8**). These expression patterns suggested that the five BnGELP proteins from region I of the zymogram in the clade 5 may have distinct roles in different tissues.

### Expression profiles of the clade 5 *BnGELP* genes in response to abiotic stresses

**Figure 7:**
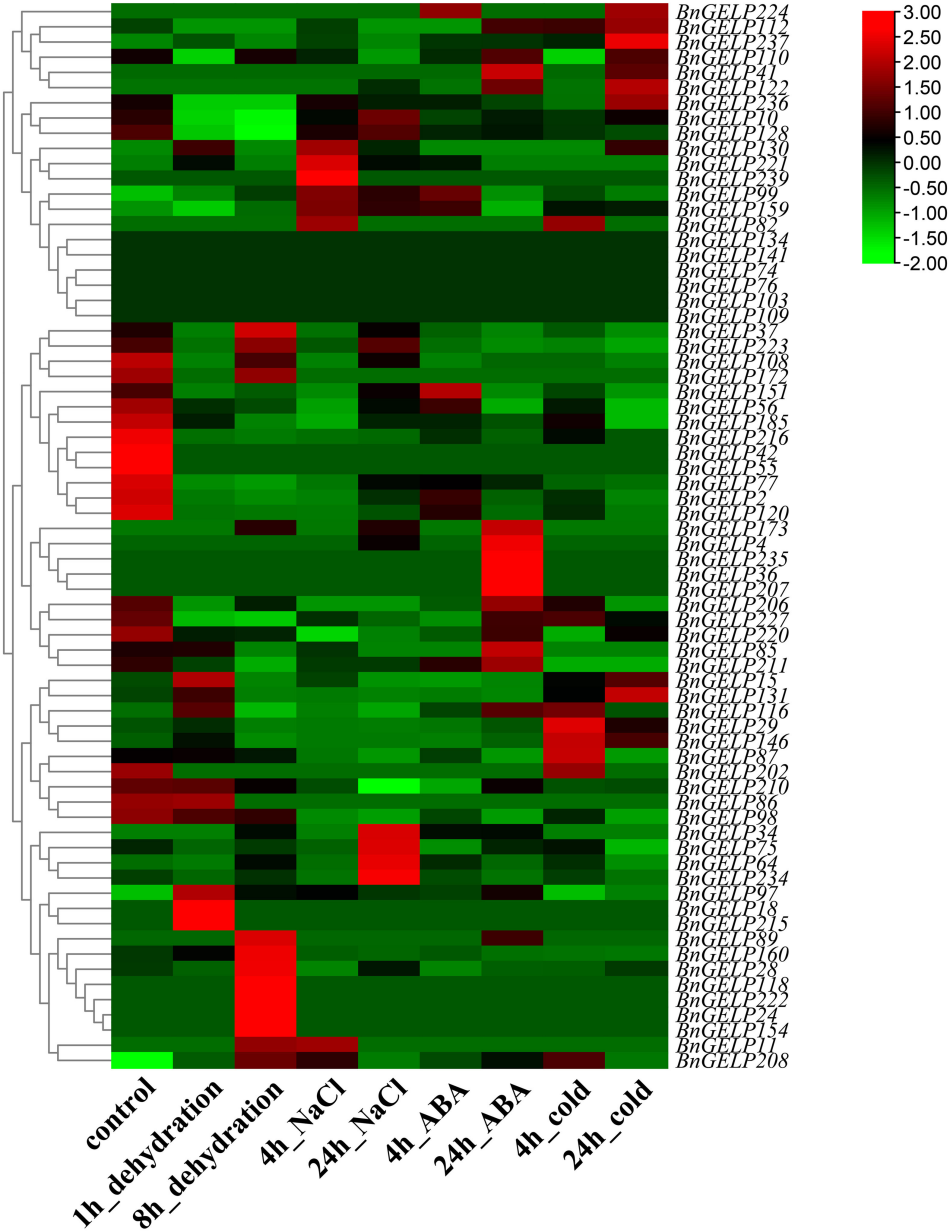
Expression profiles of the clade 5 *BnGELP* genes under different stresses. Transcript levels for the 70 *BnGELP* genes were obtained from public transcriptome data obtained from *B*. *napus* cultivar ZS11 under different abiotic stresses. Each relative expression level is equal to the mean values and transformed log_2_ values for normalization. The color scale represents relative expression levels from low (green colored) to high (red colored).

To further examine whether the expression levels of the *BnGELP* genes in the clade 5 were affected by abiotic stresses, we studied the expression profiles of all *BnGELP* genes under different abiotic stresses (dehydration, NaCl, ABA and cold conditions, **Supplementary Figure 7**) using the published RNA-seq data (Song et al., 2022). The transcript levels of most of the clade 5 *BnGELP* genes (60 out of 70) were induced in response to at least one analyzed treatment, while 11 genes were repressed by at least one analyzed treatment (**Figure 7** and **Supplementary Table 9**). Eight genes were not detected in response to any of the analyzed treatments. Moreover, some genes were significantly induced by certain treatments. For instance, *BnGELP208* was induced by all the analyzed treatments, and *BnGELP29* and *BnGELP146* were induced by cold. In contrast, the levels of *BnGELP216* and *BnGELP77* were decreased with all analyzed treatments (**Figure 7** and **Supplementary Table 9**). The expression levels of the five identified *BnGELP* genes in clade 5 (*BnGELP37, 75, 99, 159* and *216*) in response to abiotic stress were diverse. Expression of *BnGELP99* and 159 was induced by NaCl, ABA and cold, while the expression of their close paralog, *BnGELP216*, was significantly repressed in all analyzed treatments (more than 2-fold, **Figure 7** and **Supplementary Table 9**). Expression of *BnGELP37* was slightly induced by dehydration but significantly repressed by ABA and cold. However, the expression of its close paralog, *BnGELP75*, was only slightly induced by NaCl (no more than 2-fold, **Figure 7** and **Supplementary Table 9**). These expression patterns suggested that the five identified BnGELP proteins in the clade 5 may function distinctly in response to abiotic stresses.

### Analysis of *cis*-acting elements in promoters of the clade 5 *BnGELP* genes

**Figure 8:**
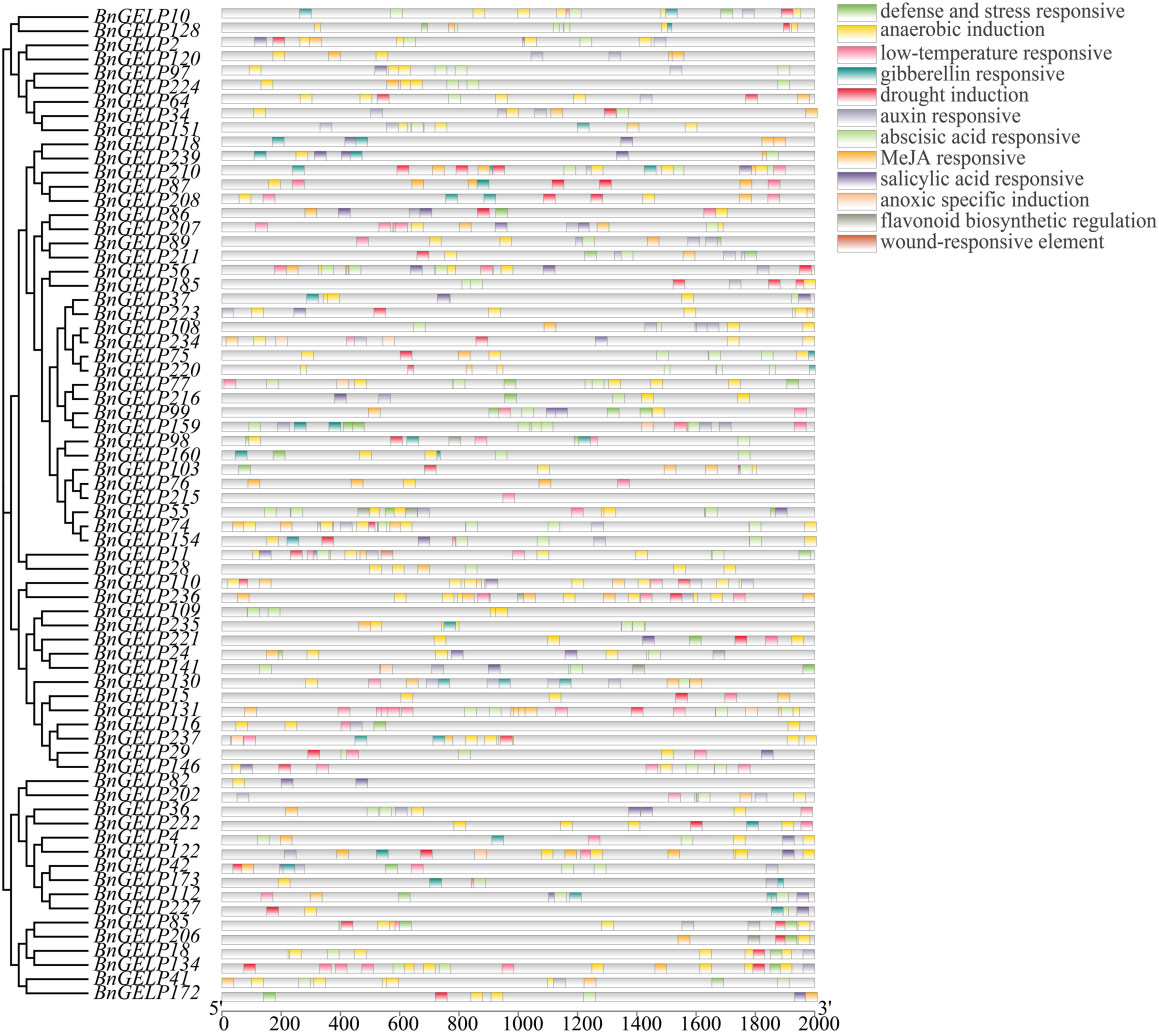
Predicted *cis*-elements in the promoter regions of the clade 5 *BnGELP* genes. The name of each regulatory element is shown on the right panel, with different colored boxes representing different *cis*-elements. The relative location of each element on each promoter can be estimated by the scale at the bottom.

The *cis*-acting elements in a promoter can provide clues about the expression profile of the corresponding gene (Shen et al., 2022). As displayed in **Figure 8**, a total of 12 *cis*-acting elements were identified in promoters of the 70 *BnGELP* genes in clade 5. There were *cis*-acting elements for anaerobic induction in 65 genes and for abscisic acid responsive in 53 genes (**Figure 8** and **Supplementary Table 10**). Forty-eight of the genes had *cis*-acting elements related to methyl jasmonate (MeJA), while 42 had elements related to low-temperature responses (**Figure 8** and **Supplementary Table 10**). The MYB binding site was present in 45 genes, with 38 that recognized by transcription factors involved in drought response and 7 for those related to flavonoid biosynthesis. Three other hormone-responsive *cis*-acting regulatory elements were detected in the promoter regions of the clade 5 *BnGELP* genes, with 41 genes carrying auxin responsive elements, 27 having gibberellin responsive elements, and 31 carrying salicylic acid responsive elements. It is worth noting that 9 out of these 12 *cis*-acting elements were found in the promoters of the five *BnGELP* genes in clade 5 (*BnGELP37, 75, 99, 159* and *216*; **Figure 8** and **Supplementary Table 10**). These included *cis*-acting elements involved in the responses to abscisic acid, anaerobic induction and low temperatures, suggesting that they may be active during low oxygen or temperature conditions and under the control of plant growth regulators, namely abscisic acid, auxin and gibberellin.

### Expression profiles of the *BnGELPs* identified by mass spectrometry in response to cold

**Figure 9:**
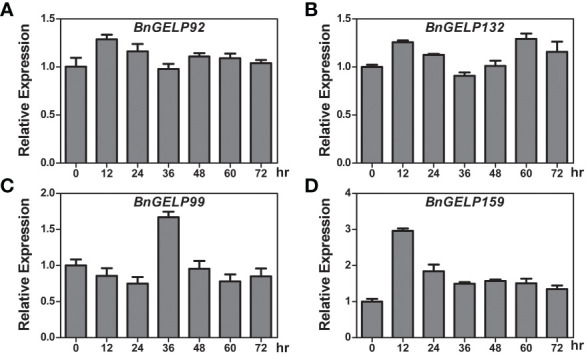
Expression of the *BnGELP* genes identified by mass spectrometry in response to cold. **(A–D)** qRT-PCR analysis of the expression of *BnGELP92, 132, 99* and *159* in response to cold treatment. Seedlings of *B*. *napus* cultivar ZS11 were placed at 4 °C and sampled at 0, 12, 24, 36, 48, 60 and 72 hours. The relative expression levels of *BnGELP92, 132, 99* and *159* were normalized to the expression of *BnACTIN*. The primers are list in **Supplementary Table 11**.

As shown in **Figure 8**, there were two putative low-temperature responsive elements detected in the promoters of *BnGELP99* and *159*. To ascertain whether cold affects the expression of the *BnGELP* genes identified by mass spectrometry, ZS11 was treated with cold, and the expression of them was investigated by qRT-PCR. Inducible expression (more than 2-fold) of *BnGELP159* was observed to occur 12 hr following cold treatment, showing a decrease to approximately 1.5-fold at 24 hr, which persisted through 72 hr (**Figure 9**). Inducible expression (more than 1.5-fold) of *BnGELP99* was observed to occur 36 hr following cold treatment (**Figure 9**). The other two identified *BnGELPs*, *BnGELP92* and *132*, do not harbor any low-temperature responsive elements in their promoters (**Supplementary Figure 8**) and neither were induced by cold (no more than 1.5-fold) during the analyzed treatment time (**Figure 9** and **Supplementary Figure 8**).

Because the expression of *BnGELP99* and *159* were affected by cold temperature and because of their relationship with the esterase isozyme activity in region I of the zymogram (**Figure 9** and **Figure 4**), we next explored whether cold could impact the esterase activity in ZS11. Cold treatment increased esterase activity in both regions I and II of the zymogram, including the first and second bands in region I (**Supplementary Figures 9**, **10**). These two bands may be related to the identified *BnGELP* genes in clade 5.

Mobilization of stored lipid and their conversion into soluble sugars (hexoses and sucrose) are crucial for the establishment of seedlings. To explore the putative interrelationships between soluble sugar content and esterase isozyme activity, accumulation of soluble sugars in different *B. napus* lines and in response to cold was determined. As expected, the soluble sugar contents are higher than control condition after 36 hr following cold treatment (**Supplementary Figure 10**). Together these results suggested that the cold-inducible expression of the identified *BnGELP* genes in clade 5 may be correlated with increasing activities of esterase isozymes by cold temperatures.

## Discussion

The hydrolysis of the lipid stored in oil bodies by esterase/lipase plays a vital role during seed germination and early post-germinative growth of rapeseed (Ding et al., 2019a; Ding et al., 2019b; Zhan et al., 2022). One class of these esterases/lipases is the GELPs, which have been shown to be involved in the hydrolysis of stored lipids in the initial stage of seed germination in rice (Dolui and Vijayaraj, 2020). GELPs have broad substrate specificity and have a high number of family members within a genome. Multiomics technologies, including genomics, transcriptomics, proteomics and metabolomics, provide opportunities to systematically explore all the genes belonging to a specified gene family within a species (Zhan et al., 2022). Numerous *GELP* family members have been identified in different plant species (Chepyshko et al., 2012; Dong et al., 2016; Lai et al., 2017; Su et al., 2020; Lv et al., 2021; Wang et al., 2021; Zhang et al., 2021; Cenci et al., 2022; Jiao et al., 2022; Song et al., 2022; Zhan et al., 2022). Recently, a high-quality genome assembly and numerous transcriptome data sets have been released for *B. napus* cultivar ZS11 (Chen et al., 2021; Liu et al., 2021), making identification and expression analysis of genes such as the *BnGELPs* more feasible.

In the present study, 240 *GDSL-*like genes were identified from *B. napus* cultivar ZS11. The *BnGELPs* were divided into 5 clades based on phylogenetic tree (C1 to C5, **Figure 1** and **Supplementary Table 2**), which is not consistent with previous reports (Volokita et al., 2011; Lai et al., 2017; Karunarathna et al., 2020; Lv et al., 2021; Cenci et al., 2022). The number of *BnGELPs* was greater than the number identified in other plant species (Volokita et al., 2011; Chepyshko et al., 2012; Dong et al., 2016; Lai et al., 2017; Su et al., 2020; Lv et al., 2021; Zhang et al., 2021; Cenci et al., 2022; Jiao et al., 2022; Song et al., 2022; Zhan et al., 2022). The BnGELP proteins were not equally distributed into the 5 different phylogenetic clades (**Figure 1**), with the two largest clades (C2 and C5) representing around 60% of the total BnGELP members (**Figure 1** and **Supplementary Table 2**), suggesting they may be highly differentiated in the *B. napus* genome. Moreover, the diversity of the molecular weight and isoelectric points of the BnGELP proteins may reflect their functional diversity, which may be attributed to differences in amino acid sequences or domains besides the conserved GDSL domains (**Figure 2** and **Supplementary Figures 4**, **5**).

GELPs have a wide range of substrate specificities and are capable of mobilizing multiple lipids, making it easy to detect their biochemical activity using a gel essay called a zymogram (Akoh et al., 2004; Zhu et al., 2017; Ding et al., 2019b; Shen et al., 2022). Ten BnGELPs were detected in region I of the zymogram of ZS11 seedling extracts (**Figure 4** and **Supplementary Figure 3**), providing important clues about the role of the BnGELPs in the regulation of seed germination *via* hydrolysis of the stored lipids. Further motif, gene expression profiles and *cis*-elements analyses of the *BnGELPs* in the clade 5 suggested that members of this clade may play different roles in different tissues and in response to different abiotic stresses. Numerous esterases/lipases have been reported to be involved in the hydrolysis of stored lipids in the initial stage of seed germination, such as serine hydrolases (SHs), GDSL-type esterases/lipases, proteases, Ser carboxypeptidases, ABHD protein, and pectin acetylesterase (Dolui and Vijayaraj, 2020). Interestingly, we also detected other esterases/lipases besides GDSL-type proteins by mass spectrometric analysis (**Supplementary Table 5**), implying that there should be more esterases/lipases involved in the hydrolysis of stored lipids in the initial stage of seed germination.

A total of 12 *cis*-acting elements were identified in promoters of the *BnGELP* genes in clade 5 (**Figure 8** and **Supplementary Table 10**), suggesting that their expression may be induced by low oxygen levels, low temperatures, and abscisic acid, auxin and gibberellin. It is worth noting that most of the clade 5 *BnGELP* genes (65 out of 70) harbored elements related to anaerobic induction. Any flooding stress during the sowing season may deprive the plant roots of oxygen, which can lead to an adjustment in the expression of genes related to seed germination and early seedling establishment, i.e., genes controlling lipid metabolism, glycolysis, energy metabolism, signal transduction, and photosynthesis. However, no detailed reports have linked the regulation of anaerobic induction and seed germination with respect to fatty acid metabolism. Moreover, a slight cold-inducible expression of *BnGELP99* and *159* was observed (**Figure 9**), which may be attributed to two *cis*-acting regulatory elements predicted to be involved in low-temperature responsiveness present in their promoters. An increase in the activity of esterase isozymes by cold was also observed (**Supplementary Figure 10**), which may corelate with other cold-inducible esterase/lipase genes besides our identified *BnGELPs*. Nevertheless, these hypotheses need to be further verified by conducting further genetic and biochemical studies.

## Conclusion

In the current study, a total of 240 *GDSL-*like genes were identified from *B. napus* cultivar ZS11. A systematic analysis of the *BnGDSL *gene family, including phylogenetic relationships, chromosomal location, gene synteny, protein and gene architectures, expression profiles in different tissues and in response to different abiotic stress, as well as *cis*-elements in the promoters, was conducted to gain insights into the evolutionary and functional characteristics of the clade 5 *BnGELPs*. Moreover, BnGELPs in clade 5 were linked to the esterase activity in an isozyme zymogram during early seedling establishment *via* mass spectrometry. In addition, expression of *BnGELP99* and *159* may be correlated with the increase in activity of esterase isozymes in response to cold.

## Data availability statement

The original contributions presented in the study are included in the article/**Supplementary Material**. Further inquiries can be directed to the corresponding authors.

## Author contributions

CX and YZ conceived the project and designed the experiment plans. YD, LX, JX, TJ, XT and YW conducted the experiments. CX and YZ analyzed the data and wrote the article. SH, WH and XZ reviewed and edited the manuscript. All authors contributed to the article and approved the submitted version.
