# Supplementary material for: Genome-wide exploration of the GDSL-type esterase/lipase gene family in rapeseed reveals several BnGELP proteins active during early seedling development
Source: Front Plant Sci. 2023 Mar 15;14:1139972. doi: 10.3389/fpls.2023.1139972 (PMC10050346; doi:10.3389/fpls.2023.1139972)
Supplement: Supplementary file 1 [file DataSheet_1.docx]

**Supplementary Figure 1**

**
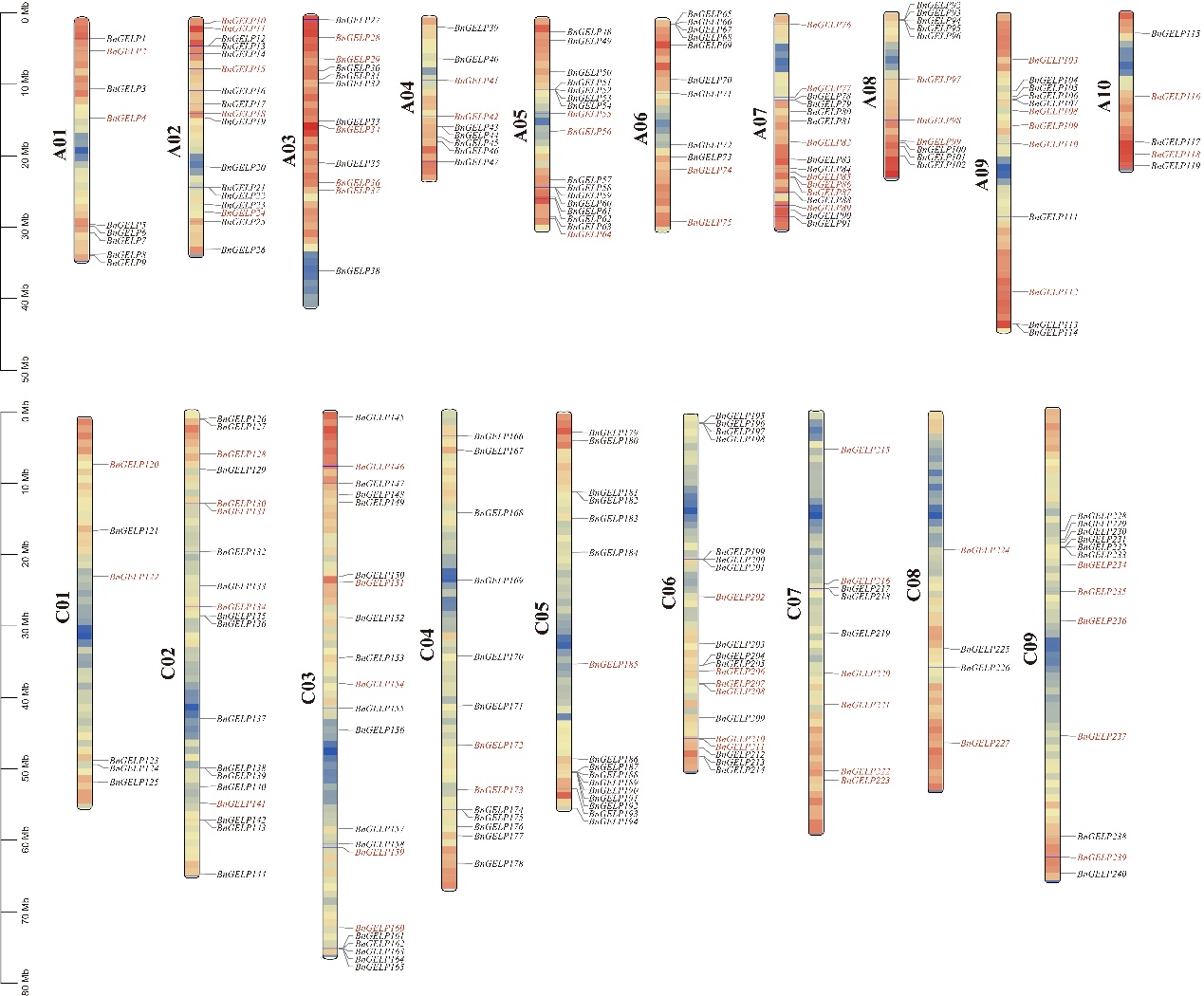
**

**Supplementary Figure 1. Distribution of the identified *BnGELP* genes on 19 chromosomes of *B. napus.*** Chromosome numbers are indicated at the top of each bar and chromosomal distances are given in Mbp at the left of each bar. The *BnGELP* genes from the C5 group are red labeled, the other members of *BnGELP* genes are labeled in black.

**Supplementary Figure 2**

**
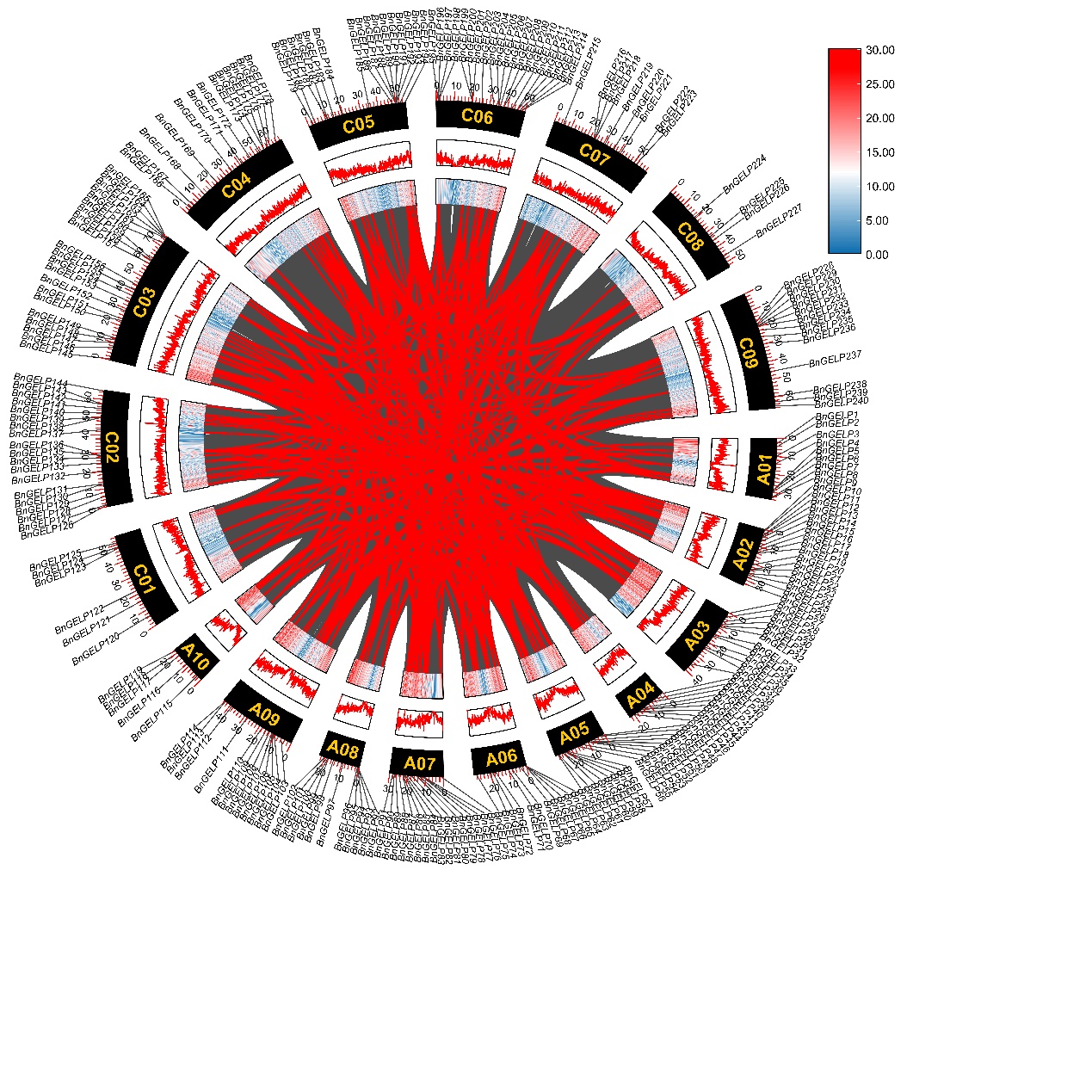
**

**Supplementary Figure 2. The synteny analysis of *BnGELP* genes in *B. napus*.**

The gray lines display all synteny blocks, and the red lines indicate the segmental duplicate gene pairs in *Brassica napus* Cultivar ZS11 genome respectively.

**Supplementary Figure 3**


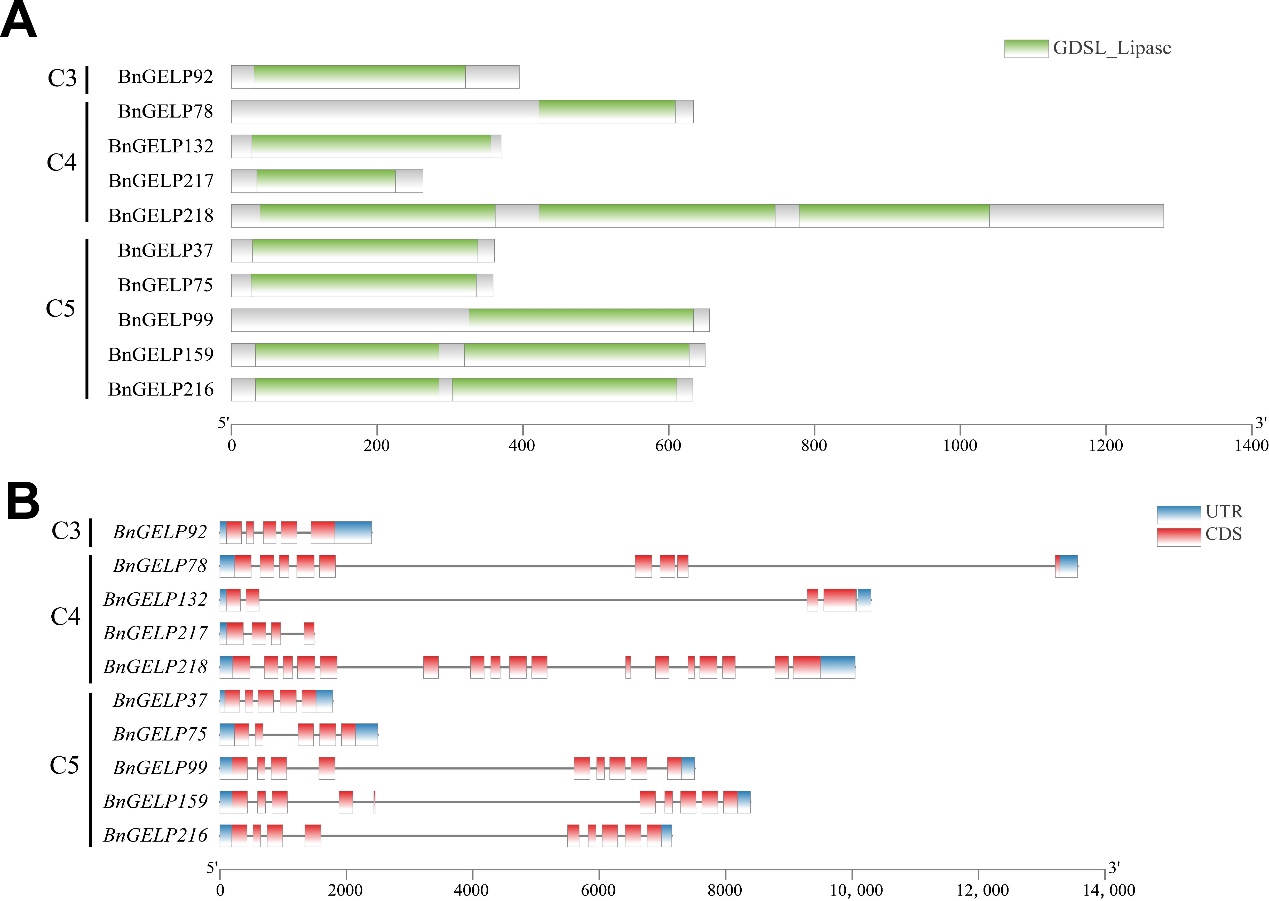


**Supplementary Figure 3**. **The** **conserved GDSL motif and gene structure of the identified BnGELPs by mass spectrometry.** (A) The conserved GDSL motifs of the identified BnGELPs are indicated with green boxes. (B) Schematic exon/intron structures of the identified *BnGELPs*. The red boxes represent exons and black lines represent introns. The UTR regions are indicated in blue boxes. The length of each CDS can be estimated by the scale at the bottom.

**Supplementary Figure 4**

**
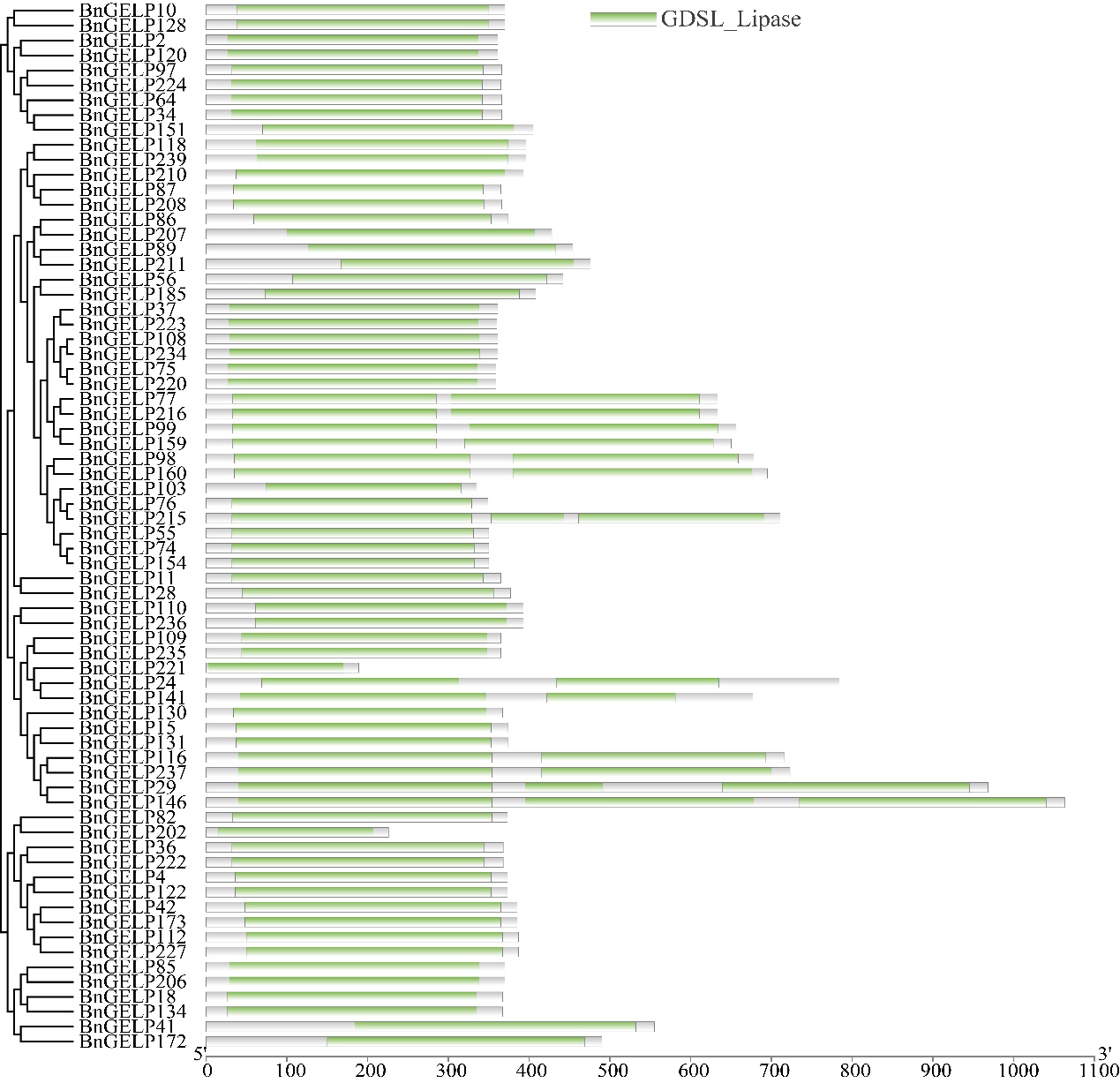
**

**Supplementary Figure 4. The conserved GDSL motif of BnGELP proteins in clade 5.**

The conserved GDSL motifs are indicated with green boxes.

**Supplementary Figure 5**

**
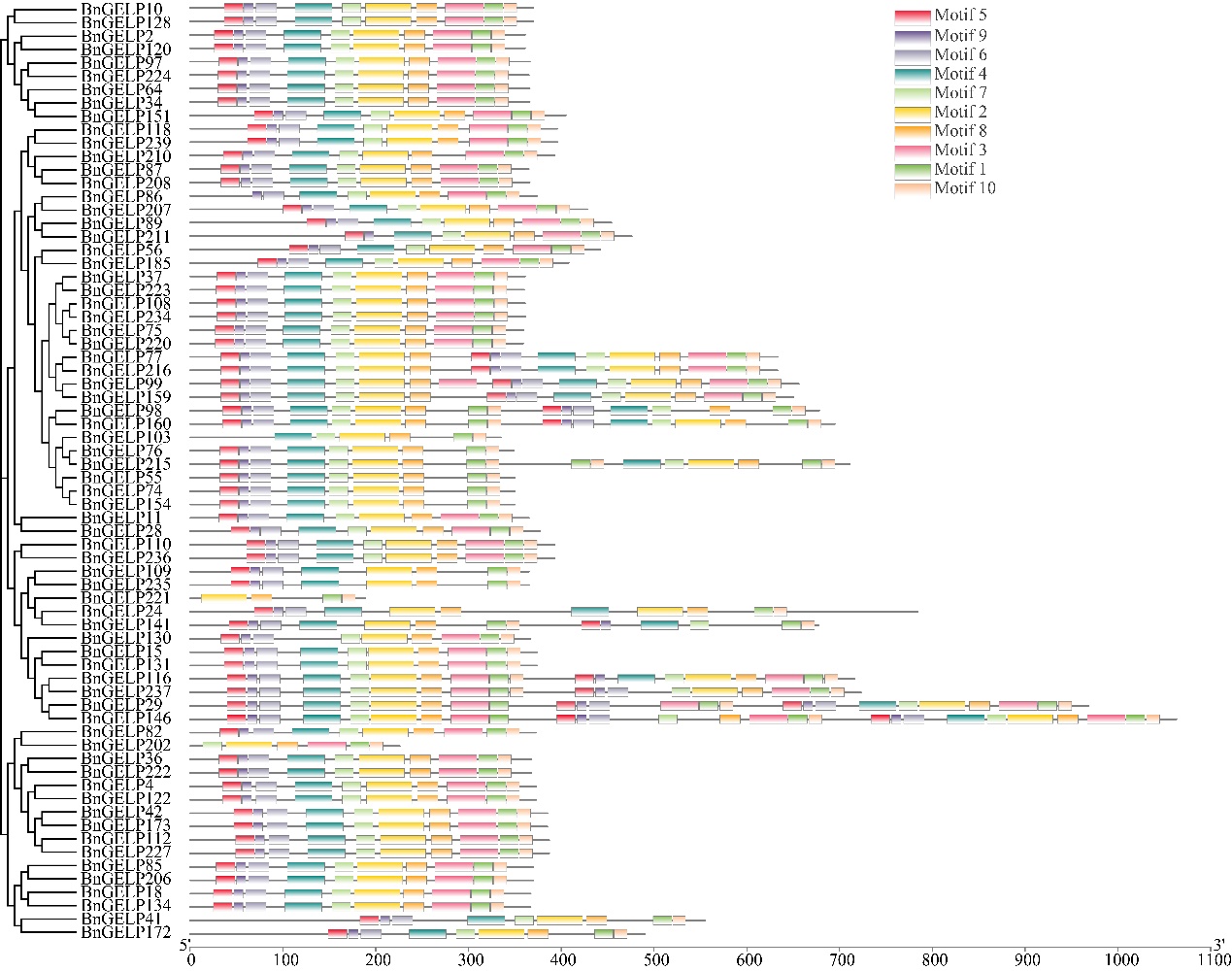
**

**Supplementary Figure 5.** **Conserved motifs of BnGELP proteins in clade 5.**

Boxes of different colors represent different motifs.

**Supplementary Figure 6**

**
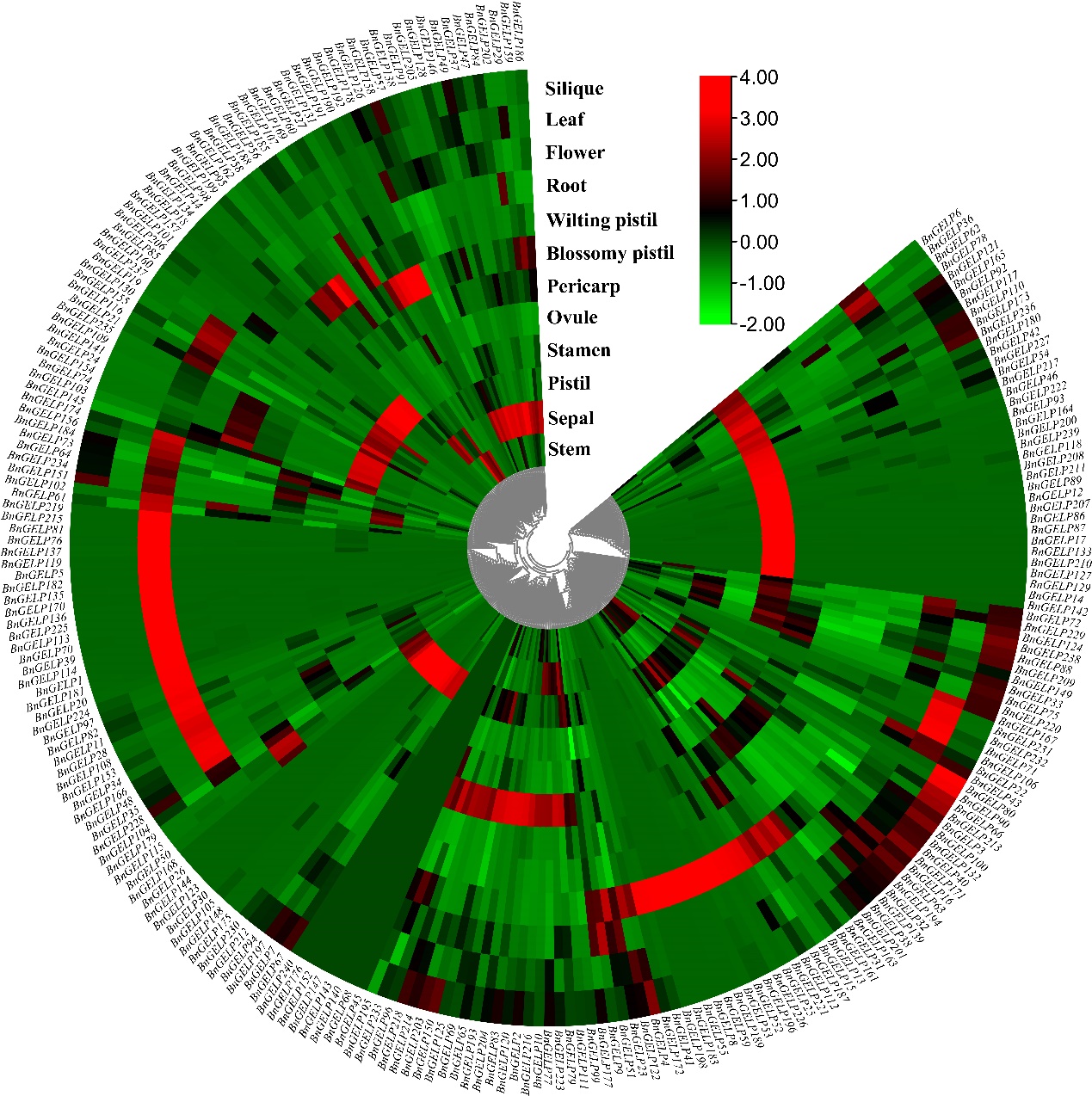
**

**Supplementary Figure 6. Expression profiles of the *BnGELP* genes in different tissues.**

The expression level is equal to the mean values and transforms log_2_ values for normalization. The color scale represents relative expression levels from low (blue colored) to high (red colored).

**Supplementary Figure 7**

**
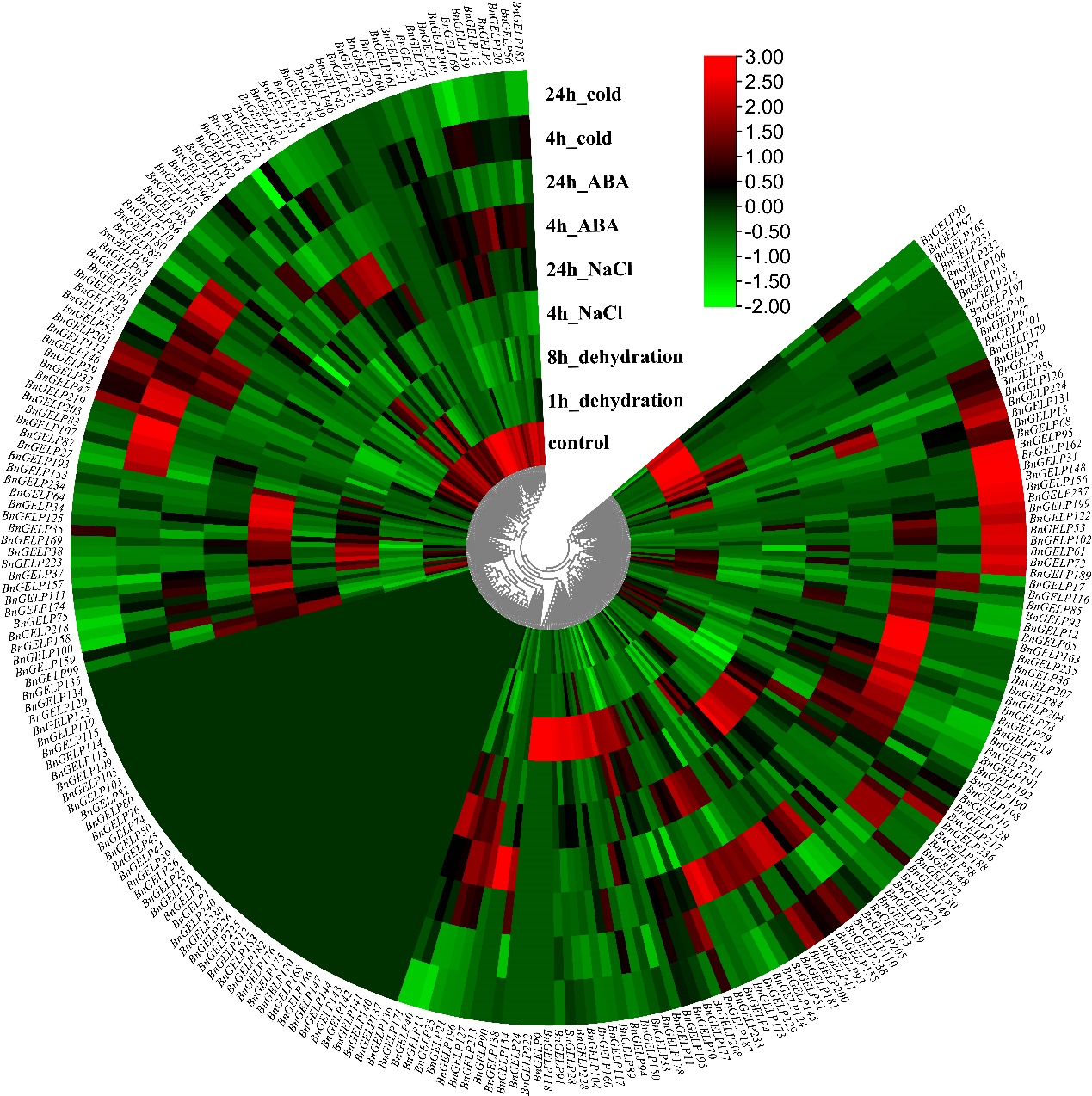
**

**Supplementary Figure 7. Expression profiles of the *BnGELP* genes under different stresses.** The expression level is equal to the mean values and transforms log_2_ values for normalization. The color scale represents relative expression levels from low (blue colored) to high (red colored).

**Supplementary Figure 8.**

**
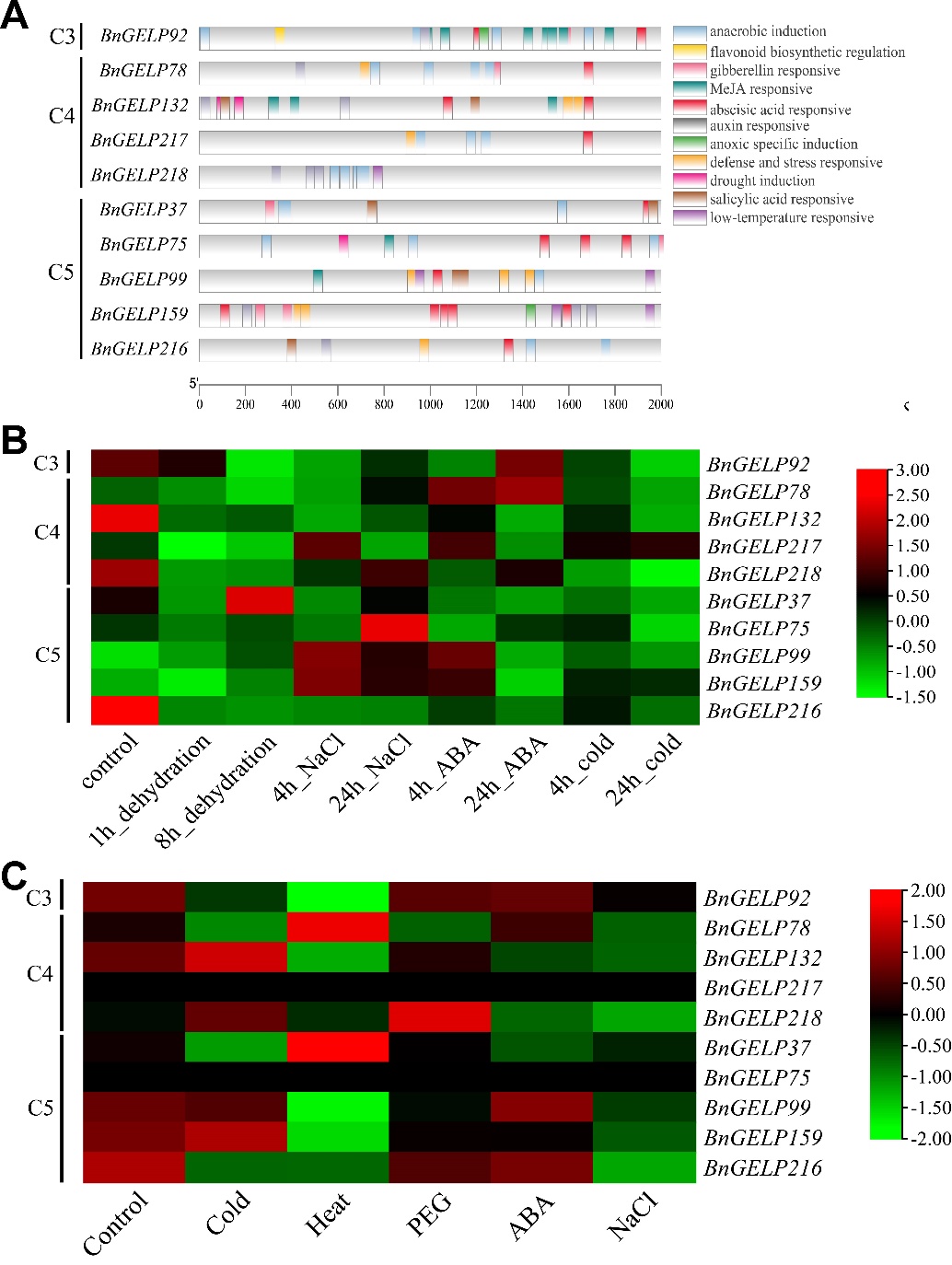
**

**Supplementary Figure 8**. **The *cis*-acting elements** **and gene expression of the identified BnGELPs by mass spectrometry under different stresses.** (A) The *cis*-acting elements found in the promoter region of the identified BnGELPs. (B) Expression (TPM) of the identified *BnGELPs* under different stresses. RNA-seq data from NGDC (accession number CA001775). (C) Expression (TPM) of the identified *BnGELPs* under different stresses. RNA-seq data was download from NCBI (accession number SRP109808).

**Supplementary Figure 9.
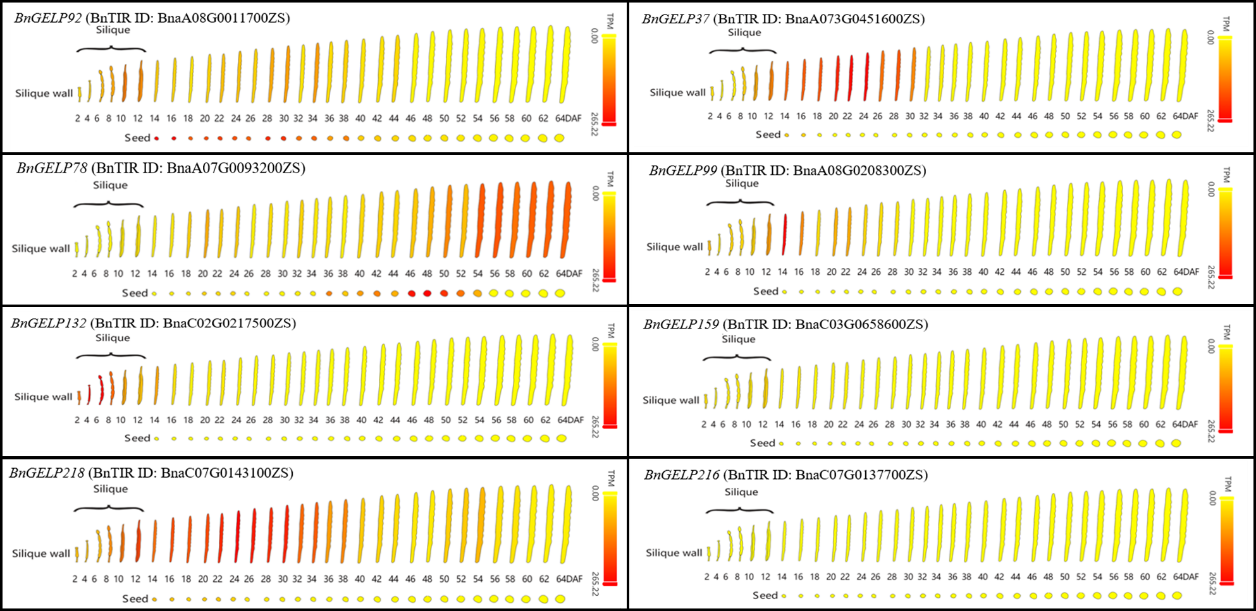
**

**Supplementary Figure 9. The expression level of the identified *BnGELP* genes in the seeds.** The expression level of the identified *BnGELP* genes in the seeds were obtained from the *Brassica napus* RNA-seq data (http://yanglab.hzau.edu.cn/BnTIR).

**Supplementary Figure 10**.

**
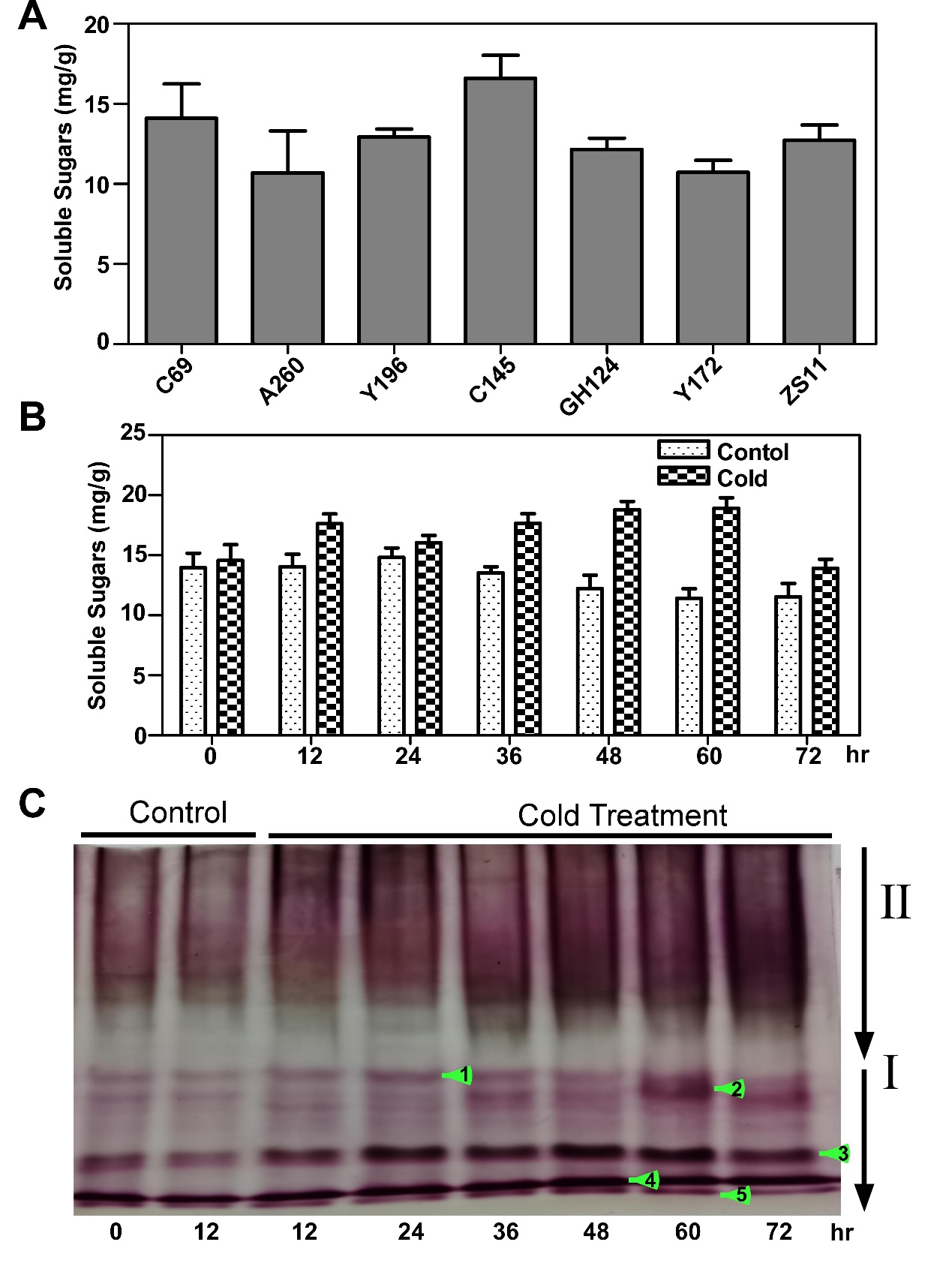
**

**Supplementary Figure 10.** **Accumulation of soluble sugars and the zymogram of esterase isozymes in response to cold.**

The level of soluble sugars (reducing and nonreducing) was determined using phenol-sulfuric acid based colorimetric method as previously described (Burbulis et al., 2011). (A) Accumulation of soluble sugars in different *Brassica napus* materials. (B) Accumulation of soluble sugars during cold treatment in *Brassica napus* cultivar ZS11. (C) The zymogram of esterase isozymes in *Brassica napus* cultivar ZS11 in response to cold.
